# Prognostic Value of FDG PET Imaging in Patients with Laryngeal Cancer

**DOI:** 10.1371/journal.pone.0096999

**Published:** 2014-05-12

**Authors:** Kazuhiro Kitajima, Yuko Suenaga, Tomonori Kanda, Daisuke Miyawaki, Kenji Yoshida, Yasuo Ejima, Ryohei Sasaki, Hirokazu Komatsu, Miki Saito, Naoki Otsuki, Ken-ichi Nibu, Naomi Kiyota, Tsutomu Minamikawa, Kazuro Sugimura

**Affiliations:** 1 Department of Radiology, Kobe University Graduate School of Medicine, Kobe, Japan; 2 Department of Radiology, Teikyo University School of Medicine, Tokyo, Japan; 3 Department of Radiology, Division of Radiation Oncology, Kobe University Graduate School of Medicine, Kobe, Japan; 4 Department of Otolaryngology-Head and Neck Surgery, Kobe University Graduate School of Medicine, Kobe, Japan; 5 Department of Medicine, Division of Medical Oncology/Hematology, Kobe University Graduate School of Medicine, Kobe, Japan; 6 Department of Oral and Maxillofacial Surgery, Kobe University Graduate School of Medicine, Kobe, Japan; Wayne State University, United States of America

## Abstract

**Background and Purpose:**

To investigate the prognostic value of ^18^F-fluorodeoxyglucose positron emission tomography (FDG-PET) in patients with laryngeal cancer.

**Materials and Methods:**

The study included 51 patients of whom 30 underwent definitive radiotherapy with or without chemotherapy and 21 underwent radical surgery with or without adjuvant chemoradiation therapy. FDG uptake by both the primary lesion and the neck node was measured using the maximum standardized uptake value (SUVmax). The effects of clinicopathological factors including primary tumor SUVmax and nodal SUVmax on progression-free survival, local control, nodal progression-free survival, and distant metastasis-free survival were evaluated using the log-rank test and Cox method.

**Results:**

The median duration of follow-up was 48.6 months (range 8 to 82.1 months). Univariate analysis showed that nodal SUVmax, N status, and tumor TNM stage were significantly associated with recurrence, whereas primary tumor SUVmax, age, treatment strategy and T status were not. Multivariate analysis demonstrated that only the nodal SUVmax was a significantly unfavorable factor for progression-free survival (p = 0.029, hazard ratio 0.54, 95% CI 0.38-0.87) and nodal progression-free survival (p = 0.023, hazard ratio 0.51, 95% CI 0.34-0.81). ROC curve analysis and log-rank test showed that patients with a high nodal SUVmax (≧4) had a significantly lower progression-free survival rate than those with a low SUVmax (<4; p<0.0001).

**Conclusions:**

The pretreatment SUVmax of nodal disease in patients with laryngeal cancer is prognostic for recurrence.

## Introduction

Various treatment strategies are used to improve outcome in patients with squamous cell carcinoma of the head and neck. Selection of appropriate treatment strategies and prognostication remain difficult for clinicians, despite careful evaluation of clinical factors, TNM staging, and anatomic subsite. Identification of novel pretreatment imaging biomarkers that would potentially predict long-term outcome would be clinically significant.

With the use of ^18^F-fluorodeoxyglucose (FDG), a glucose analog, positron emission tomography (PET) allows non-invasive assessment of glucose metabolism in a wide variety of tumor types including head and neck cancer. Tumor FDG uptake has been associated with various cellular characteristics such as cell viability and proliferation activity [1], [2]. Thus, analyses of metabolic parameters, which are independent of morphologic changes, are expected to offer an important opportunity to predict individual tumor behavior.

Although several studies have found that metabolic activity evident FDG-PET in patients with a variety of head and neck cancer subtypes (i.e. nasopharynx, oropharynx, hypopharynx, larynx, oral tongue, gum, buccal mucosa, mouth floor) has prognostic significance [3], [4], the prognostic value of FDG-PET for squamous cell carcinoma of head and neck cancer remains controversial. Moreover, there is no information on the prognostic value of FDG-PET in only laryngeal cancer, and it remains uncertain whether FDG-PET in patients with laryngeal cancer actually yields prognostic information. We performed a retrospective review of 51 patients with laryngeal cancer who underwent FDG-PET at initial presentation to determine whether FDG uptake by the primary tumor and neck lymph nodes is correlated with recurrence.

## Materials and Methods

### Patient

Written prior informed consent to undergo FDG-PET imaging and receive treatments was obtained from all patients. The institutional review board (Kobe University Hospital, Japan) approved this retrospective study (No 1401); patient informed consent for inclusion in this study was waived. To protect patient privacy, we removed all identifiers from our records at the completion of our analyses. Our primary selection criteria for patients included those who underwent FDG-PET scan as a pretreatment staging examination at our institution within 2 weeks before treatment for biopsy proven squamous cell laryngeal carcinoma, between October 2006 and September 2011. On the basis of these primary criteria, 60 consecutive patients were selected. Of these, 9 were excluded because of (a) a follow-up duration of less than 6 months (n = 6), and (b) presence of distant metastasis (n = 3). A total of 51 patients (46 males, 5 females; average age at diagnosis 69.1 years, range 56–86 years) meeting the eligibility criteria for this study were included in the analysis.

Pretreatment systematic evaluations were performed along with a routine physical examination, laryngoscopy and tissue biopsy, serum chemistry, chest radiography, contrast-enhanced CT or MRI of the head and neck, and FDG-PET scan. Clinical staging and treatment choices were decided using the information derived from these examinations at the Head and Neck Cancer Board conference of Kobe University Hospital which consisted of head and neck surgeons, radiation oncologists, medical oncologists and radiologists.

Clinical assessment of prognostic factors was performed retrospectively in all 51 patients with laryngeal cancer, in a subgroup of 30 patients who underwent definitive radiotherapy (RT) with or without chemotherapy (RT group), and in a subgroup of 21 patients who underwent radical surgery and neck dissection with or without adjuvant chemoradiation therapy (surgery group). Subsequent follow-up included physical examination, laryngoscopy, contrast-enhanced CT, and FDG-PET.

### FDG-PET study

All whole-body FDG-PET scans were acquired with a PET scanner (Philips Allegro, Philips Medical System, Best, the Netherlands) that provided 45 trans-axial images at 4-mm intervals over a distance of 18.0 cm. After at least 6 h of fasting, patients received an intravenous injection of 222 to 333 MBq (6 to 9 mCi) of FDG. After positioning the patient, a static emission scan was performed with 2.5 to 3 min of acquisition in each bed position, covering the upper thigh to the ear with a total of 9–10 bed positions. Then, a transmission scan using a ^137^Cs ring was performed over the same area for 23 s per bed position. Three-dimensional acquisition was performed and PET images were reconstructed using an ordered-subset expectation maximization iterative reconstruction algorithm (RAMLA). The field of view and pixel size of the reconstructed images were 57.6 cm and 4.0 mm, respectively, with a matrix size of 128×128.

After the FDG-PET scan had been completed, patients were moved to the CT room. The CT device was a multi-detector row CT system with an acceleration voltage of 120 kVp and a current of 80 mA. Both reconstructed PET and CT data were transferred to a workstation running viewing-dedicated software (Syntegra; SUN Microsystems, Milpitas, CA, USA) to create fused PET and CT images.

### Image analysis

PET images were retrospectively interpreted by two experienced nuclear medicine physicians. For semiquantitative analysis of FDG uptake, regions of interest (ROIs) were defined on the target lesions (primary lesion and neck lymph node) on the transaxial PET images. The maximum standardized uptake value (SUV) was calculated for quantitative analysis of tumor FDG uptake, as follows:
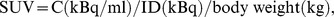
where C is the tissue activity concentration measured by PET and ID is the injected dose.

For nodal disease, the highest SUVmax was used for subsequent correlation with clinical outcomes.

### Statistical analysis

The actuarial progression-free survival (PFS), local control (LC), nodal progression-free survival (NPFS), and distant metastasis-free survival (DMFS) rates were calculated using the Kaplan-Meier method. The duration was calculated from the initial date of treatment to the date of an event or the last follow-up visit. PFS was defined as absence of death due to any cause or recurrence. LC was defined as only primary site control. NPFS was defined as any regional nodal failure after treatment as an event. DMFS was defined as the absence of any distant metastasis.

Survival data were analyzed using Kaplan–Meier plots and the log-rank test. The prognostic value of individual variables was evaluated using Cox proportional hazards logistic regression. We determined the statistically significant SUV cutoff value for survival analysis using the log-rank test and receiver operating characteristic (ROC) curve analysis.

Univariate Cox proportional hazards modeling was used to quantify the risk for recurrence of the following variables: age, treatment strategy, T status, N status, tumor TNM stage, primary tumor SUVmax, and nodal SUVmax. Subsequently, the significant or borderline univariate variables (p<0.1) were entered into multivariate analysis. The results from the Cox models were expressed as hazard ratios with 95% confidence intervals, and p values of <0.05 were considered to indicate statistical significance. All analyses were performed using SAS software version 9.2 (SAS Institute, Cary, NC).

## Results

### Patient characteristics

Patient demographics and clinicopathologic variables are demonstrated in Table 1. With regard to the distribution of TNM stages in the 51 patients, eight were at stage I, 17 were at stage II, 12 were at stage III, and 14 were at stage IV.

**Table 1:** Patient characteristics.

	RT group	Surgery group	Total
Characteristics	n = 30	n = 21	n = 51
Median age, years (range)	67 (58–83)	69 (56–86)	69 (56–86)
Sex: male/female	29/1	17/4	46/5
T status			
T1	7	2	9
T2	18	4	22
T3	5	7	12
T4	0	8	8
N status			
N0	27	11	38
N1	1	2	3
N2	1	8	9
N3	1	0	1
TNM stage (AJCC)			
I	7	1	8
II	15	2	17
III	6	6	12
IV	2	12	14
Primary tumor SUVmax			
Median (range)	2.85 (1.2–8.52)	8.6 (3.6–16.65)	4.25 (1.2–16.65)
Nodal SUVmax			
Median (range)	1.45 (0.8–9.29)	2.0 (1.0–14.76)	1.75 (0.8–14.76)

RT: radiotherapy.

n: number of patients.

AJCC: American Joint Committee on Cancer.

SUV: standardized uptake value.

In the RT group (n = 30), 20 patients received only RT at doses of 66.0 to 74.4 Gy and 10 received RT (66.0-73.2 Gy) concomitant with chemotherapy, generally two or three cycles of cisplatin with or without continuous infusion of 5-flurouracil and/or docetaxel. In the surgery group (n = 21), all patients underwent radical surgery with neck dissection. Moreover, 4 received adjuvant chemoradiation therapy and 1 received radiotherapy.

### Prognostic factors in the patients overall

After a median follow-up of 48.6 months for the patients overall, 11 (21.6%) of the 51 patients had recurrence. Among these 11 patients, four developed local recurrence, two neck nodal recurrence, and five lung metastasis. The median overall follow-up duration was 53.5 months (range 17.6 to 82.1 months) for the 40 patients without recurrence, and 21.3 months (range 8.0 to 43.4 months) for the 11 patients with recurrence at follow-up.

**Figure 1:**
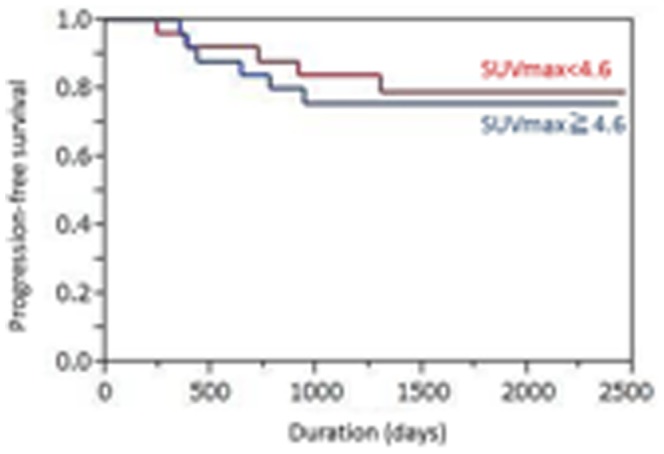
The high SUVmax (≧4.6) subgroup showed a slightly shorter median progression-free survival time than the low SUVmax (<4.6) subgroup (42.7 vs. 57.3 months; p = 0.66).

**Figure 2:**
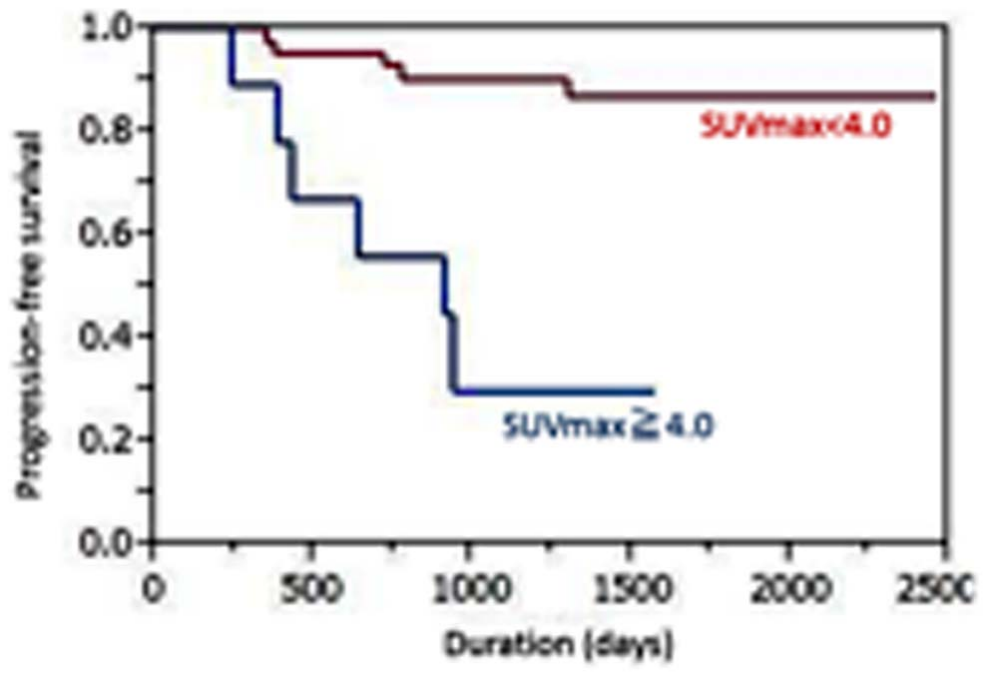
The high SUVmax (≧4.0) subgroup showed a significantly shorter median progression-free survival time than the low SUVmax (<4.0) subgroup (30.4 vs. 52.2 months; p<0.0001).

**Figure 3:**
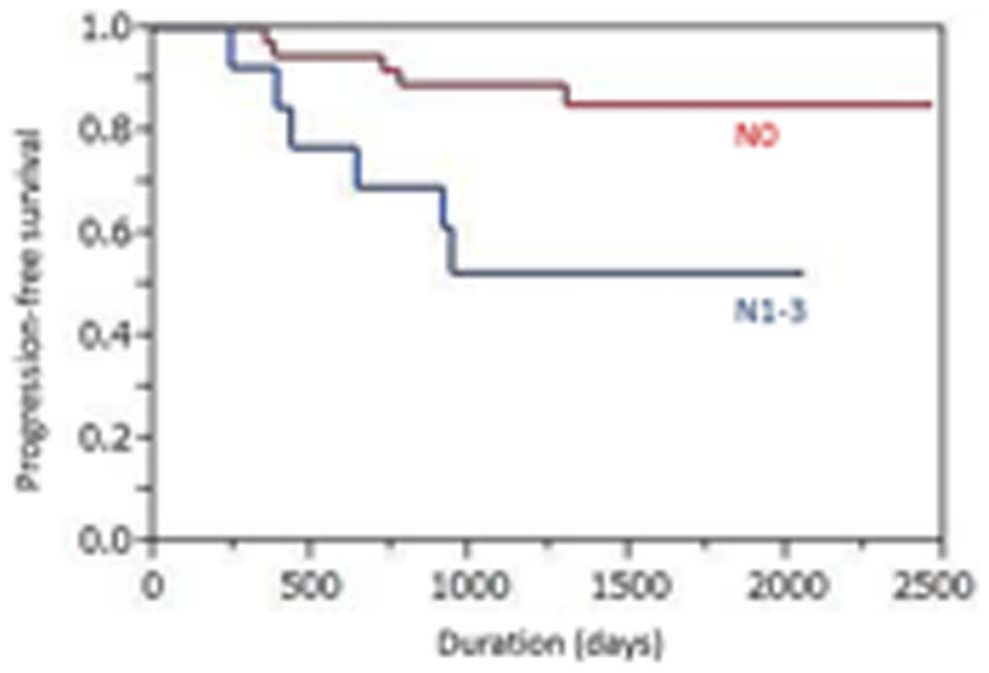
The positive lymph node status (N1-3) subgroup showed a significantly shorter median progression-free survival time than the N0 subgroup (31.3 vs. 50.9 months; p = 0.0099).

**Figure 4:**
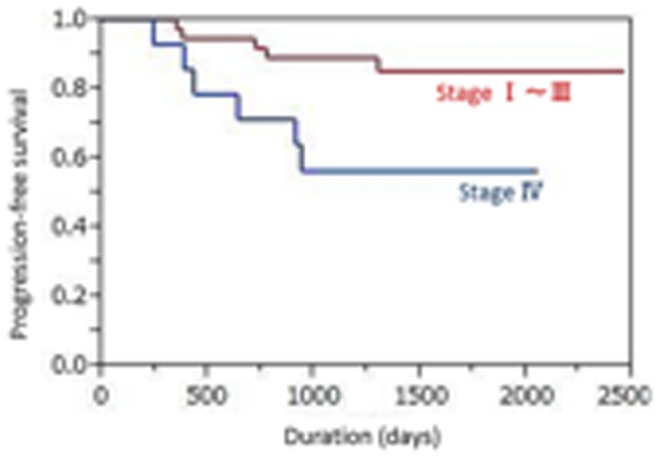
The high stage (stage IV) subgroup showed a significantly shorter median progression-free survival time than the lower stage (stage I-III) subgroup (35.1 vs. 54.3 months; p = 0.015).

The median primary tumor SUVmax was 4.25 (range 1.2–16.65). Using best discriminative cut-off for the primary tumor SUVmax (4.6) to establish two groups based on ROC curve analysis, the high SUVmax (≧4.6) subgroup showed a shorter median PFS time than the low SUVmax (<4.6) subgroup, but the difference did not reach statistical significance (42.7 vs. 57.3 months; p = 0.66) (Fig 1). The 4-year PFS rates were 44.0% versus 53.8%, respectively. The median nodal SUVmax was 1.75 (range 0.8–14.76). Using the best cut-off for nodal SUVmax (4.0) based on the ROC curve analysis, the high SUVmax (≧4.0) subgroup showed a significantly shorter median PFS time than the low SUVmax (<4.0) subgroup (30.4 vs. 52.2 months; p<0.0001) (Fig 2). The 4-year PFS rates were 22.2% versus 54.8%, respectively. Univariate analysis showed that nodal SUVmax (p<0.0001), N status (p = 0.0099, Fig 3), and tumor TNM stage (p = 0.015, Fig 4) were significantly related to PFS, whereas primary tumor SUVmax (p = 0.66), age (p = 0.11), treatment strategy (p = 0.71), and T status (p = 0.53) were not (Table 2). Multivariate analysis showed that only nodal SUVmax (risk ratio 0.54, 95% confidence interval [CI] 0.38–0.87, p = 0.029) was an independent predictor of PFS.

**Table 2:** Univariate analysis of clinicopathological factors associated with clinical outcome in the patients overall (n = 51).

Characteristics	n	p-value (log-rank)			
		PFS	LC	NPFS	DMFS
Age at diagnosis (years)					
<65	18	0.11	0.42	0.26	0.88
≥66	33				
Primary tumor SUVmax					
<4.6	26	0.66	0.38	0.96	0.16
≥4.6	25				
Nodal SUVmax					
<4.0	42	<0.0001	0.44	0.0006	<0.0001
≥4.0	9				
Treatment					
RT group	30	0.71	0.55	0.78	0.37
Surgery group	21				
T status					
T1–2	31	0.53	0.65	0.69	0.28
T3–4	20				
N status					
N0	38	0.0099	0.29	0.011	0.0018
N1–3	13				
NM stage (AJCC)					
Stage I–III	37	0.015	0.29	0.016	0.0037
Stage IV	14				

n: number of patients.

PFS: progression-free survival.

LC: local control.

NPFS: nodal progression-free survival.

DMFS: distant metastasis-free survival.

SUVmaximum: maximum standardized uptake value.

RT: radiotherapy.

AJCC: American Joint Committee on Cancer.

As shown in Table 2, no factors were found to affect LC. Nodal SUVmax, N status, and tumor TNM stage were significantly related to NPFS and DMFS, whereas primary tumor SUVmax, age, treatment strategy, and T status were not. Multivariate analysis showed that only nodal SUVmax (risk ratio 0.51, 95% CI 0.34–0.81, p = 0.023) was an independent predictor of NPFS.

### Prognostic factors in the RT group

Six (20.0%) of the 30 RT patients suffered recurrence: local recurrence in three, neck nodal recurrence in one, and lung metastasis in two. The median overall follow-up duration was 53.5 months (range 17.6 to 82.1 months) in the 24 patients without recurrence, and 25.0 months (range 8.0 to 43.4 months) in the six patients with recurrence at follow-up.

The median SUVmax values for the primary tumor and neck nodes were 2.85 (range 1.2–8.52) and 1.45 (range 0.8–9.29), respectively. Using a best discriminative SUVmax cut-off of 4.0 for the primary tumor, the high SUV (≧4.0) subgroup showed a shorter median PFS time than the low SUV (<4.0) subgroup, but the difference did not reach statistical significance (38.6 vs. 57.3 months; p = 0.63). The 4-year PFS rates were 37.5% versus 54.5%, respectively. Using a best cut-off nodal SUVmax value of 4.0, the high SUVmax (≧4.0) subgroup showed a significantly shorter median PFS time than the low SUVmax (<4.0) subgroup (19.2 vs. 50.9 months; p<0.0001). The 4-year PFS rates were 0% versus 53.6%, respectively. Univariate analysis showed that nodal SUVmax (p<0.0001), N status (p = 0.018), and tumor TNM stage (<0.0001) were significantly related to PFS, whereas primary tumor SUVmax (p = 0.63), age (p = 0.31), and T status (p = 0.12) were not (Table 3). Multivariate analysis revealed no factors that were related to PFS.

**Table 3:** Univariate analysis of clinicopathological factors associated with clinical outcome in the RT group (n = 30).

Characteristics	n	p-value (log-rank)			
		PFS	LC	NPFS	DMFS
Age at diagnosis (years)					
<65	13	0.31	0.73	0.34	0.20
≥66	17				
Primary tumor SUVmax					
<4.0	22	0.63	0.33	0.075	0.46
≥4.0	8				
Nodal SUVmax					
<4.0	28	<0.0001	0.78	<0.0001	0.0045
≥4.0	2				
T status					
T1–2	25	0.12	0.28	0.77	0.14
T3–4	5				
N status					
N0	27	0.018	0.64	0.0007	0.0040
N1–3	3				
NM stage (AJCC)					
Stage I–III	28	<0.0001	0.79	<0.0001	0.0045
Stage IV	2				

RT: radiotherapy.

n: number of patients.

PFS: progression-free survival.

LC: local control.

NPFS: nodal progression-free survival.

DMFS: distant metastasis-free survival.

SUVmaximum: maximum standardized uptake value.

AJCC: American Joint Committee on Cancer.

As shown in Table 3, none of the examined factors affected LC. Nodal SUVmax, N status, and tumor TNM stage were significantly related to NPFS and DMFS, whereas primary tumor SUVmax, age, treatment strategy, and T status were not. Multivariate analysis showed that only nodal SUVmax (risk ratio 0.48, 95% confidence interval [CI] 0.32–0.78, p = 0.018) was an independent predictor of NPFS.

### Prognostic factors in the surgery group

Five (23.8%) of the 21 surgery patients suffered recurrence: local recurrence in one, neck nodal recurrence in one, and lung metastasis in three. The median overall follow-up duration was 55.4 months (range 29.6 to 80.8 months) in the 16 patients without recurrence, and 14.2 months (range 11.5 to 31.3 months) in the five patients with recurrence at follow-up.

The median SUVmax values for the primary tumor and neck nodes were 8.6 (range 3.6–16.65) and 2.0 (range 1.0–14.76), respectively. Using a best discriminative SUVmax cut-off of 9.8 for the primary tumor, the high SUVmax (≧9.8) subgroup showed a shorter PFS time than the low SUVmax (<9.8) subgroup, but the difference did not reach statistical significance (42.7 vs. 47.3 months; p = 0.50). The 4-year PFS rates were 50.0% versus 46.7%, respectively. Using a best nodal SUVmax cut-off of 4.0, the high SUVmax (≧4.0) subgroup showed a significant shorter median PFS time than the low SUVmax (<4.0) subgroup (30.7 vs. 60.5 months; p = 0.013). The 4-year PFS rates were 28.6% versus 57.1%, respectively. Univariate analysis showed that only nodal UVmax (p = 0.013) had a significant relationship with PFS, whereas primary tumor SUVmax (p = 0.50), age (p = 0.17), T status (p = 0.56) N status (p = 0.12), and tumor TNM stage (p = 0.29) did not (Table 4). Multivariate analysis showed that none of the examined factors affected PFS.

**Table 4:** Univariate analysis of clinicopathological factors associated with clinical outcome in the surgery group (n = 21).

Characteristics	n	p-value (log-rank)			
		PFS	LC	NPFS	DMFS
Age at diagnosis (years)					
<65	5	0.17	0.58	0.56	0.27
≥66	16				
Primary tumor SUVmax					
<9.8	15	0.50	0.53	0.13	0.74
≥9.8	6				
Nodal SUVmax					
<4.0	14	0.013	0.48	0.17	0.0037
≥4.0	7				
T status					
T1–2	6	0.56	0.11	0.56	0.87
T3–4	15				
N status					
N0	11	0.12	0.34	0.32	0.049
N1–3	10				
NM stage (AJCC)					
Stage I–III	9	0.29	0.25	0.41	0.12
Stage IV	12				

n: number of patients.

PFS: progression-free survival.

LC: local control.

NPFS: nodal progression-free survival.

DMFS: distant metastasis-free survival.

SUVmaximum: maximum standardized uptake value.

AJCC: American Joint Committee on Cancer.

As shown in Table 4, none of the factors examined were related to LC. Nodal SUVmax and N status were significantly related to DMFS, whereas primary tumor SUVmax, age, T status and tumor TNM stage were not. Multivariate analysis showed that none of these factors was related to DMFS.

## Discussion

To our knowledge, this is the first study to have evaluated the clinical usefulness of FDG-PET for providing prognostic information on patients with only squamous cell laryngeal carcinoma. Although several studies have demonstrated that metabolic activity evident on FDG-PET has prognostic significance in patients with a variety of head and neck cancer subtypes (i.e. nasopharynx, oropharynx, hypopharynx, larynx, oral cavity) [3], [4], the various primary tumor burdens may differ, thus affecting FDG uptake, treatment response and survival, all of which could cause potential biases.

In our series, nodal SUVmax rather than the primary tumor, was significantly associated with PFS and NPFS. A similar tendency has also been reported in three previous studies [5]–[7]. Demirci et al. [5] demonstrated that a nodal SUV exceeding 4.45 posed a greater risk of recurrence in 64 patients with various head and neck cancers including those of the nasopharynx (n = 29), larynx (n = 16), oropharynx (n = 13), or hypopharynx (n = 6) treated by radiotherapy or surgery. Inokuchi et al. [6] reported that a nodal SUV exceeding 6.0 posed a greater risk of poor outcome (in terms of DFS, NPFS, and DMFS) in 178 patients with various head and neck cancers including those of the oral cavity (n = 61), nasopharynx (n = 38), oropharynx (n = 34), hypopharynx (n = 27), larynx (n = 13), or nasal sinus (n = 5) treated using chemoradiation. They also showed that among the patients with a greater nodal SUVmax (>6.0), those who underwent planned neck dissection had longer NPFS than those in the observation only group. Kubicek et al. [7] showed that a nodal SUV exceeding 10.0 posed a greater increased risk of distant failure in 212 patients with various head and neck cancers including those of the oropharynx (n = 89), larynx (n = 54), oral cavity (n = 29), salivary gland (n = 13), nasal sinus (n = 9), hypopharynx (n = 3), or unknown primary (n = 5) managed using various types of therapy. We suggest that high FDG uptake in neck nodes is correlated with poor outcome, and that such patients should receive more aggressive treatment combinations.

The prognostic value of primary tumor SUVmax in patients with head and neck cancer remains controversial, and many reports have indicated that it has positive [8] or negative [9] associations with outcome. Allal et al. [8] demonstrated that a primary tumor SUV exceeding 4.76 posed a greater risk of poor outcome in 120 patients with various head and neck cancers including those of the oropharynx (n = 46), oral cavity (n = 32), larynx (n = 26), hypopharynx (n = 13), or unknown primary (n = 3) managed by radiotherapy or surgery. Tang et al. [9] showed that primary tumor SUV was not significantly associated with survival in 83 patients with various head and neck cancers including those of the oropharynx (n = 45), nasopharynx (n = 22), hypopharynx (n = 8), oral cavity (n = 4), larynx (n = 4), or unknown primary (n = 2) managed by radiotherapy.

As is the case for all novel biomarkers, there are also potential limitations and concerns regarding the widespread applicability of SUV. For example, it has been demonstrated that SUV varies with respect to time after injection of FDG [10]. The exact plasma glucose value may also affect SUV, even in the absence of frank hyperglycemia/diabetes [11]. The body habitus of the patient (independent of his/her actual weight) may also affect SUV, because fatty tissue shows relatively low FDG uptake. Finally, there are a number of technical factors that can affect SUV, as has been reviewed in a comprehensive editorial by Keyes [12]. These factors include the recovery coefficient (the ratio of the measured activity of a ROI relative to its true activity) and partial volume averaging, which are affected by individual nuances of the hardware and software of the PET scanner, the size and geometry of the lesion, and respiratory motion [13].

Moreover, although convenient to measure and widely used, SUVmax has a disadvantage. It is a single-pixel value representing the most intense FDG uptake in the tumor and may not represent total uptake for the whole tumor mass, as well as being vulnerable to statistical noise, which might explain the current results. Recently, volume-based metabolic parameters measured by FDG-PET have emerged as new prognostic factors. Metabolic tumor volume (MTV) is defined as the volume of FDG activity in the tumor, and total lesion glycolysis (TLG) as the summed SUV within the tumor. Unfortunately, in our series, we were unable to measure MTV and TLG due to technical limitations of the PET machine. This is one of several limitations to our present study.

There were other limitations. First, it was a retrospective study performed at a single institution with a relatively small number of patients, especially in surgery group. Second, FDG-PET was not performed initially for every patient with laryngeal cancer, as only selected patients were referred for PET scanning. The use of FDG-PET in only selected patients might have introduced bias and influenced the results, which may therefore not be generalizable to all subjects. Third, the volumetric analyses such as MTV and TLG were not undertaken because of PET technological problem. Finally, we were unable to analyze overall survival because there were only three disease-related deaths among the study population.

## Conclusions

Laryngeal cancer patients showing high FDG uptake in neck nodes should be considered at increased risk of poor outcome and may benefit from more aggressive multimodality treatment combinations. These results remain to be confirmed in a larger prospective and more homogeneous study.
